# Mapping upper-limb motor performance after stroke - a novel method with utility for individualized motor training

**DOI:** 10.1186/s12984-017-0335-x

**Published:** 2017-12-06

**Authors:** Orna Rosenthal, Alan M. Wing, Jeremy L. Wyatt, David Punt, R. Chris Miall

**Affiliations:** 1 School of Psychology, University of Birmingham, B15 2TT, Birmingham, UK; 2 School of Computer Science, University of Birmingham, B15 2TT, Birmingham, UK; 3 School of Sport, Exercise and Rehabilitation Sciences, University of Birmingham, B15 2TT, Birmingham, UK

**Keywords:** Stroke, rehabilitation, Motor assessment, Robot-assisted therapy, Upper-limb movements, Reaching task

## Abstract

**Background:**

Chronic upper limb motor impairment is a common outcome of stroke. Therapeutic training can reduce motor impairment. Recently, a growing interest in evaluating motor training provided by robotic assistive devices has emerged. Robot-assisted therapy is attractive because it provides a means of increasing practice intensity without increasing the workload of physical therapists. However, movements practised through robotic assistive devices are commonly pre-defined and fixed across individuals. More optimal training may result from individualizing the selection of the trained movements based on the individual’s impairment profile. This requires quantitative assessment of the degree of the motor impairment prior to training, in relevant movement tasks. However, standard clinical measures for profiling motor impairment after stroke are often subjective and lack precision. We have developed a novel robot-mediated method for systematic and fine-grained mapping (or profiling) of individual performance across a wide range of planar arm reaching movements. Here we describe and demonstrate this mapping method and its utilization for individualized training. We also present a novel principle for the individualized selection of training movements based on the performance maps.

**Methods and Results:**

To demonstrate the utility of our method we present examples of 2D performance maps produced from the kinetic and kinematics data of two individuals with stroke-related upper limb hemiparesis. The maps outline distinct regions of high motor impairment. The procedure of map-based selection of training movements and the change in motor performance following training is demonstrated for one participant.

**Conclusions:**

The performance mapping method is feasible to produce (online or offline). The 2D maps are easy to interpret and to be utilized for selecting individual performance-based training. Different performance maps can be easily compared within and between individuals, which potentially has diagnostic utility.

**Electronic supplementary material:**

The online version of this article (10.1186/s12984-017-0335-x) contains supplementary material, which is available to authorized users.

## Background

Impaired upper-limb (UL) function is one of the most common consequences of stroke [1–3], which can severely hamper activities of daily living and reduce quality of life. Certain intervention methods can promote some recovery of UL motor function though their outcome shows high variability and depends on the intensity (repetition) of the intervention [4–9].

Robotic assistive technologies can be beneficial for improving clinical scores of UL motor impairment [9, 10], by allowing intensive training [9, 11–14]. However, currently there is no consistent evidence for the effectiveness of robot-assisted UL therapy for improving daily living activity [15]. One possibility is that the tasks performed with robotic assistance do not generalise to everyday tasks. Another possibility is that the tasks are not optimised for the trained individuals. Currently, in robot-assisted therapy the set of practiced movements are usually pre-determined, with limited regard to the motor profile of the individual (e.g. ‘centre-out’ point-to-point reaches, or forearm pronation/supination, wrist extension/flexion [16–18]). However, the effectiveness of training for motor recovery is likely to depend on the difficulty to perform the task due to motor impairment [19]. For example, training focused on unimpaired movements or on tasks that are either too easy or too difficult is likely to contribute relatively little to motor learning and recovery [19–21]. An advantage of the robot-mediated approach is that it allows the collection of various accurate and real-time data about motor performance that would be potentially useful for individualized adjustments of the therapy; e.g. selection of training tasks based on the profile of motor performance. Yet, prescribing training conditions based on a motor performance profile requires characterising motor performance across a range of movement conditions for each individual. Here we present a novel computerised method for systematically mapping individuals’ UL motor performance (or impairment) across a wide range of robot-mediated reaching movements. The map can then serve as a basis for individualised and performance-based selection of training movements.

For optimal utilization of a motor performance map, the mapped metrics should reflect basic components of sensorimotor control, so that the map can be directly linked to processes underlying the movements (e.g. muscle activity and movement representation). Continuous metrics, allowing smoothing and interpolation from tested movements to neighbouring untested regions are also valuable. Accordingly, our mapping of reaching performance is done across the two dimensions of target location (in angular coordinates relative to a central position) and of prescribed starting location (again in angular coordinates relative to the selected target, which indicates the dictated movement direction). The range of target and start locations tests both postural and movement-related aspects of motor control, respectively. Importantly, muscle activation patterns and population neural activity in the motor-related cortices show tuning to one or both task dimensions [22–25], and behavioural studies support the essential underlying role of these parameters in planning of reaching movements [26, 27].

Of course, the usefulness of a motor performance map for prescribing performance-based training also depends on an appropriate principle for the selection of movements to be practiced. Here we demonstrate the utility of our mapping method for individualized task selection based on a principle which we term “steepest gradients” (SG), although the motor performance map can be the basis for alternative task selection principles. The SG principle is founded on the idea that training with tasks performed with an intermediate range of difficulty would allow more improvement and learning-induced plasticity, compared to training with very difficult or easy tasks [19, 28] .

Here we report the details of the mapping methods, and show its efficacy in portraying relevant motor impairment patterns for individual subjects. We also briefly demonstrate its utility for individually-tailored selection of practiced movement using the SG principle. However, our evidence for the utility and benefit of the mapping method for individualizing UL robot-mediated rehabilitation after stroke will be reported in subsequent publications.

## Methods

### Ethics, consent and permissions

In this report we demonstrate examples of the principle and utility of the novel performance and impairment mapping method using the data of two adults with UL hemiparesis due to a stroke (in the chronic stage), who participated in an on-going study. This study was approved by the Science, Technology, Engineering and Mathematics Ethical Review Committee of the University of Birmingham (ERN_09-528). Prior to their participation, participants received detailed information about the study and provided written consent.

### Procedure and Task

The method of performance mapping presented here is applied to a robot-assisted reaching task, although the mapping principles do not depend on a specific robot-assisted algorithm and could be used with other forms of motor performance data.

The participants took part in multiple sessions of robot-assisted start-to-target reaching exercises where different sessions served different purposes. Both participants first completed parameter tuning and performance mapping sessions (see below). Participant 2 also took part in 15 training sessions, which we report to demonstrate our method of performance-based training.

**Fig. 1:**
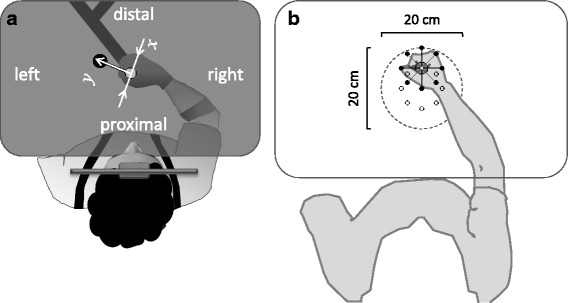
Schematic description of the experimental setting (top view). **a** The participant held the handle of a robotic manipulandum (indicated onscreen by a red disc; not shown), which allowed planar reaching movements from a start position (white onscreen disc (here gray) to a target position (blue onscreen disc; here black) and provided assisting and guiding forces as needed. Hand’s grip was maintained via a special glove and the forearm was supported against gravity (not shown). The participant leaned his/her head against a headrest, maintaining upright seating posture (ensured using a harness). The horizontal display occluded the hand and the manipulandum from vision. The start-to-target axis (y) and its perpendicular axis (x) correspond to the axes of the assisting and guiding forces, respectively, which were provided during the arm movement as needed by the robot. Adapted from Howard et al. (2009). **b** The reaching workspace used for mapping performance. The locations of the 8 targets, used in the mapping sessions, are indicated by small open circles. An example of the arm posture when the hand located at the 90^o^ target is shown. Participants were tested with 5cm reaches to each target from 8 start locations (indicated, for the example target, by small black dots). The dashed circle indicates the extent of the mapped workspace. The drawing reflects the actual relationship of target and start locations and arm posture, based on a photograph taken with a healthy participant

During each of the task sessions the participants held a robotic manipulandum (vBot[29]) with the arm supported against gravity using a SaeboMass device (http://www.saebo.com/). At the beginning of each trial, the robot gently moved the participant’s hand towards a start position and maintained the hand there until a blue target appeared on the display. The vBot then released hold of the hand and the participant was asked to reach the target, as accurately as possible, within an individually-set allotted time (Fig. 1a). Then the target disappeared and an animated explosion feedback (not shown) informed the proximity of the final hand position to the target (see the additional file for full task details [Additional file 1, ‘Task and settings’]). Assistive and guiding forces were provided by the vBot during the reach, as needed (see below and the additional file [Additional file 1, ‘Robot assistance algorithm’]).

### Robot assistance

During each trial the vBot provided assistance and guidance as needed, employing a revised version of the robot-assist algorithm that has been developed for MIT-MANUS[16], and that has been reported to improve clinical scores for UL motor performance [30, 31]. Since the focus of this report is on the mapping procedure, we provide only a summary of the robot-assistance algorithm. Briefly, throughout each trial, assisting (*Assist*) forces were provided in the direction of the start-target axis and aimed to promote smooth forward movement within the allotted time and/or to impede abnormally fast rebound movements due to high muscle tone or impaired motor control (see Fig. 3b and the additional file [Additional file 1, ‘Robot assistance algorithm’]). Guiding (*Guide*) forces were provided perpendicularly to the start-target axis and acted to oppose deviation from the intended movement direction.

**Fig. 2:**
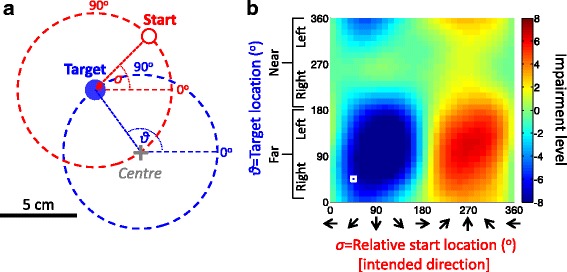
Mapping impairment across the workspace. **a** The reaching movement workspace for mapping motor performance is defined by a range of targets and their corresponding relative start locations. All targets are located on a blue circle around a pre-defined central location on the display (illustrated by a gray cross, neither circle or cross was visible to participants). The possible start locations also lie on an unseen circle around each target (illustrated for one target by a red dashed circle; see also Figure 1b). The location of the target and relative start location are defined in angular coordinates. An example of a pair of target location ϑ =135^o^ (blue disk) and relative start location σ=45^o^ (red circle) is shown. Note that each relative start location corresponds to an intended movement direction, and that each pair of target and start location coordinates is a point on a 2D map. **b** An illustration of an impairment map. The impairment level (here in normalized units) in each movement task was mapped in terms of its target and relative start condition. The coordinates of the example movement in panel a are shown on the map by a white square. Note that the map coordinates are radial and NOT spatial coordinates (x,y)

### Parameter tuning

The allotted movement time governing the *Assist* forces and the stiffness parameters governing the *Guide* force were individually set in an initial parameter tuning session, but were then maintained at fixed levels throughout the rest of the protocol. For the tuning session, subsets of the test movements were presented in short blocks to allow staircase-wise parameter adjustment, based on the participant’s performance. Usually, tuning of the assisted parameters approached a plateau within a single session. In rare cases there was a need of an extra tuning session. For further details of this tuning, see the additional file [Additional file 1, ‘Robot-assistance parameter tuning session’].

### Performance mapping principle

To create a performance (or impairment) map, participants were tested with 5cm planar start-to-target reaches in different directions and locations. Eight possible targets were located a fixed 5cm distance from a pre-set central point, and each target could be approached from eight different start locations (Figs 1b and 2a). Note that a start location defined in angular coordinates relative to the target also corresponds to the intended reach direction. Using fixed movement displacement and fixed radius of target distance allows the creation of a 2D map for any scalar metric of motor performance or impairment, by specifying each movement condition in terms of the angular coordinates of the target location and the relative start location (Fig. 2b). Each movement from a start location to a target location becomes a single point on the 2D map. In principle, other aspects of movement could be added to create 3- or high-dimensional maps. For example, distance could be a third variable. However, we restrict our work to two dimensions, movement direction and target location.

**Fig. 3:**
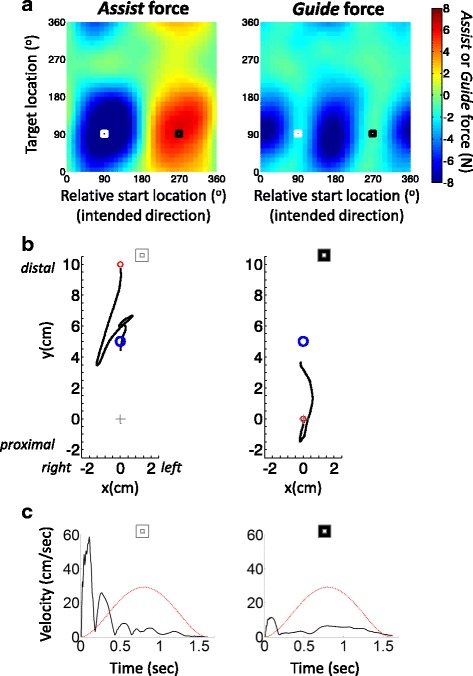
Impairment maps. **a** An example of *Assist* and *Guide* force maps shown for one UL hemiparetic stroke participant (participant 1; allotted movement time=1.6 sec, guiding stiffness=2.7 N•cm^-*1*^). Negative (*blue*) and positive (*red*) values denote forces that are provided to oppose and assist movement direction, respectively. The white and black squares refer to the conditions shown on the left and right of panel b, respectively. **b** Examples of reach trajectories for two individual movements performed by Participant 1. The left panels correspond to the map coordinates in panel a labelled by a white square. The right panels correspond to the maps’ coordinates labelled by the black square. The red and blue circles denote start and target location, respectively. The grey cross indicates the centre of the workspace. Note that the two conditions differ in start location but share same target location. **c** example of speed time series for the same example movements shown in in panel b The black line indicates actual reaching speed and red line indicates ideal minimum jerk speed profile computed according to the allotted movement duration and displacement [36]. Note the very fast rebound-like movement in the left graphs of panels b and c. The later correction of the movement (upwards towards the target; panel b) is a result of the opposing *Assist* force provided by the robot. Note also the very slow movement in the right graphs (panels b and c) and the failure to reach the target even with robot assistance, due to high elbow flexor tonus and, potentially, weak elbow extensors

The performance mapping procedure usually took a single session of less than one hour to complete. During a mapping session, motor performance was assessed across all 64 reaching movement conditions, defined by all combinations of 8 target locations and 8 relative start locations (Fig. 2a). The targets were located equidistant on an imaginary circle of 5cm radius centred 24 cm in front of the headrest and specified by their angular coordinates. For each target, the 8 possible start locations were arranged equidistant on an imaginary circle of 5 cm radius, centred on the particular target (Figs. 1b, 2a). In each performance mapping session, the 64 conditions were repeated 5 times in a pseudo-random order (320 trials in total).

Because the robot assistance applied during reaching confounds many of the usual kinematic measures of motor performance (e.g. reach errors, movement time, peak velocity) we chose to map the movement impairment principally in terms of the levels of assisting and guiding forces that were provided. Specifically, the *Assist* parameter is defined as the root-mean-square force (*F*_*y*_), which was provided during the attempt to move along the start-to-target axis (*y*):

$$ Assist=\mathit{\operatorname{sign}}\left({F}_y\right)\sqrt{\frac{1}{N}\sum \limits_{i=1}^N\kern1em {F}_y{(i)}^2} $$ , where $$ \mathit{\operatorname{sign}}\left({F}_y\right)=\left\{\begin{array}{c}-1,\;\sum \limits_{i=1}^N{F}_y(i)<0\\ {}1,\;\sum \limits_{i=1}^N{F}_y(i)\ge 0\end{array}\right. $$

(*i*=1..N indicates the time points during the allotted movement time.)

The *Guide* parameter is defined as the root-mean-square of the guiding force (*F*_*x*_) provided normal to the start-target axis (*x*):

$$ Guide=-\sqrt{\frac{1}{N}\sum \limits_{i=1}^N{F}_x{(i)}^2} $$.

Negative values mean that the force direction opposed the hand’s movement direction. This was always the case for the *Guide* parameter, as it always opposed lateral deviation. Positive values of the *Assist* parameter indicate that the force was provided in the direction of the hand movement towards the target, while negative *Assist* values mean that the force impeded abnormally very fast movements.

Separate 2D impairment maps were created for the *Assist* and *Guide* data, collected across all the 64 conditions. The raw data from all 320 trials was interpolated using a Gaussian process regression toolbox (www.GaussianProcess.org/gpml; version 3.1 for Matlab) to create a higher resolution and smoother map (with 32 x 32, or 1024 locations). Note that both the target and relative start locations were specified in angular coordinates (*θ* and *σ*, respectively). To allow Gaussian process regression and interpolation across the full angular range, these coordinates were transformed to a pair of sine and cosine coordinates, creating a 4-dimensional space (i.e., [*ϑ*, *σ*] → [sin*ϑ*, cos*ϑ*, sin*σ*, cos*σ*]). The interpolated 4D data were then transformed back to be graphically presented in original 2D angular coordinates (Fig. 2b). Finally, note that, for cross-participant comparison, the spatial coordinates of participants with left arm motor impairment should be mirror-flipped before the mapping.

### Performance-based selection of training sets

Once a motor performance/impairment map has been created for a participant, it can be used for prescribing performance-based training tasks. Here we apply a “steepest gradients” (SG) principle for selection of training tasks that is based on performance gradients across the 2D map (Fig. 5a, b). This principle is based on our suggestion that training with movements represented on the performance map at regions of transition (steep gradient) from low to high difficulty levels would be most beneficial .

**Fig. 4:**
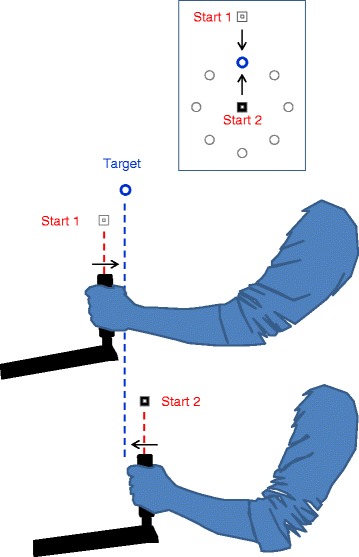
Example of arm postures at start location. Side view illustrations of the arm posture at the start location of the two reaching movements shown in Fig. 3. Both reaches are to same target location (90^o^; see inset). The start conditions are labelled by a small white and black square (see also Fig. 3). The arrows indicate the required movement direction. The upper start posture (‘start 1’; *White Square*), which corresponds to the left example in Fig. 2b and c, involves greater elbow extension. Thus, in the case of high elbow extensor tone (as for the participant in Fig. 3), holding the hand at that start position would lead to tension directed towards the target and to a fast rebound-like movement (in this example towards the target), after the release of the robotic hold. The bottom posture (‘start 2’; *black square*) corresponds to the right example in Fig. 3b and c. Here the reaching movement requires elbow extension. Hence, high elbow extensor muscle tone and weak elbow flexors would impede the movement. The illustrations are based on photographs of a healthy representative participant

The SG principle for selection of movement conditions could be based on the gradients across many kinds of performance map. However, the *Assist* and *Guide* parameters are unsuitable for gradient-based analysis because they are not sensitive to unimpaired motor performance, i.e. when no assistance is provided and hence these parameters are limited to zero. Hence, for movement selection we mapped performance based on two performance measures, PM2 and PM3, which have been in used in other studies on robot-assisted rehabilitation [16], as they provide unbounded measures of motor performance. Briefly, PM2 and PM3 measure the ability to move and aim, relative to their expected performance criteria, which are set individually in the initial tuning session (see Additional file 1, ‘Robot-assistance parameter tuning session’). Positive and negative PM values indicate performance better and worse than criterion, respectively. Individually set performance criteria are, for PM2, the allotted movement time, and for PM3, the tolerated deviation value. For further details see the additional file [Additional file 1, ‘PM2 and PM3 parameters’]. The smoothed and interpolated PM maps were created using the same procedure used for the *Assist* and *Guide* impairment maps. While PM2 and PM3 provide continuous metrics suitable for the SG-based selection procedure, they are not directly comparable across individuals; PM3 is also a kinematic measure that can be confounded by the robot assistance. Hence we use the PM maps for SG selection, but use the *Assist* and *Guide* force maps to compare motor impairment (for further explanation see Additional file 1, ‘PM2 and PM3 maps’).

To apply the SG principle to select task conditions, 2D gradients were computed across each PM map using the Matlab function ‘*gradient’* and a subset of 102 of the total of 1024 conditions per map was selected, corresponding to map locations with the top 10% of its steepest gradients. From each of these two sets of 102 training conditions, trials were pseudo-randomly selected. Finally, the number of training conditions selected from each map (PM2 or PM3) was weighted in proportion to the mean performance over the worst 25% of each map, such that more training conditions were selected based on the map that showed worse motor performance [Additional file 1, ‘Impairment-based proportion of movement selection’]. In principle, very good or bad performance might produce a flat map, without gradient. In practice we only encountered this for unimpaired participants, and then selected movements at random; we do not think it an issue for use even with mildly impaired Individuals.

### Performance-based training sessions

All participants attended an initial robot parameter tuning session, followed by a first mapping session. For participant 2, these initial sessions were followed by a 5-week period of training with 3 training sessions per week, using the performance-based selection of movement conditions. At the end of each training week, participant 2 completed an additional testing session and updated PM2 and PM3 maps were created and served for re-selecting movements for the following week, again based on the SG principle. Hence the training selection varied week-by-week, as the maps were updated. A final post-training mapping session served for evaluating training outcome.

## Results (examples of utilizing the mapping method)

The main aim of this report is to present the principles and methods of mapping UL motor performance and of selection of training conditions based on such maps. Therefore, at this stage we only present here examples of maps of two participants from our on-going study, to demonstrate their utility. The results of our full randomized and controlled study and the assessment of the benefit of the steepest gradients training principle will be detailed elsewhere.

### Impairment maps

Figure 3a  presents an example of the *Assist* and *Guide* maps from an individual with severe right arm hemiparesis due to a left hemisphere stroke 4 years previously (participant 1; UL Fugl-Meyer (F-M) score 11/66), who’s elbow flexor muscles show high tone (Modified Ashworth Scale score 2). The *Assist* map clearly illustrates two regions of high motor impairment, with opposite polarities. The small white and black squares mark two movement examples, near the centres of the two high-impairment regions. In both movements, the target coordinate is 90^o^, indicating this participant’s difficulty to reach far targets located in the body midline (see Fig. 1b). The starting direction of the two examples are 90^o^ (white square) and 270^o^ (black square), indicating inward and outward movements along the body midline; Fig. 4 indicates the arm posture at the start of these two movements.

**Fig. 5:**
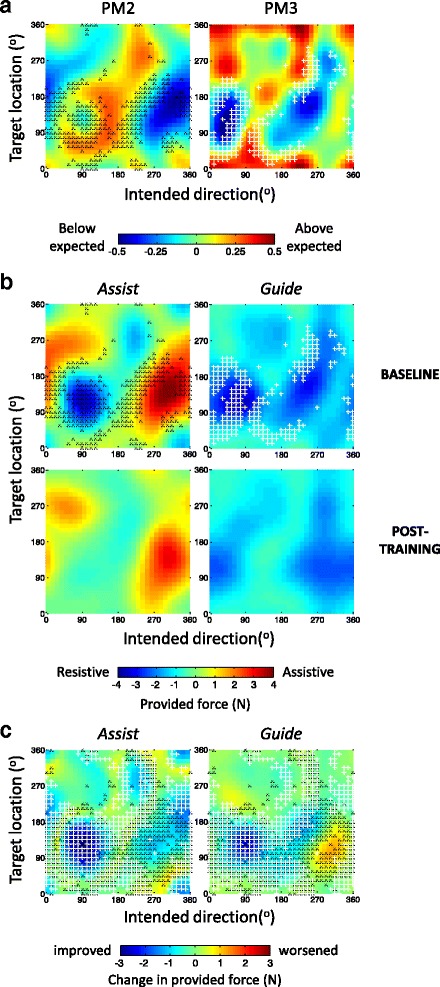
Impairment-based training. **a** Examples of selected reaching training conditions for Participant 2 (allotted movement time: 1.1 sec; guiding stiffness: 2.0 N•cm^-*1*^). The conditions for the first week of training were selected based on the PM2 and PM3 maps of the baseline (pre-training) mapping session. The selected conditions (small black ‘x’ and white ’+’ symbols, superimposed in the PM2 and PM3 maps, respectively) are within regions of 10% steepest map gradients. The selected conditions of both maps were used in the actual training. **b** The effect 5 weeks of training (3 session per week) on *Assist* and *Guide* force maps of participants 2 (left) and 3 (right). The *Assist* and *Guide* maps at the baseline session are shown at the upper row, with the selected conditions, corresponding to panel a. The lower row presents the corresponding post-training maps. **c ** Learning maps – the difference between baseline and post-training sessions per the *Assist* and *Guide* maps of each participant. Improvement is depicted as negative values. The selected training conditions (PM2-based and PM3-based combined) in all the training sessions are superimposed on the maps (small black ‘x’ and white ‘+’ symbols, respectively). The sets of training conditions were re-selected for each week based on the impairment map of the previous week

The positive red region of the *Assist* map reflects very slow outward movements that required considerable assistance (~6N) towards the target. An example from one trial, for the movement marked by small black square in Fig. 2a, is shown in Fig. 3b and c, on the right. Movement velocity is shown in Fig. 3c. Notice the very slow speed, relative to the expected by minimum jerk speed profile. The negative, blue, region of the *Assist* map reflects abnormally fast, rebound-like inward movements, which required strong robotic restraining forces to dampen the speed and to oppose target over-shooting. The left graphs of Fig. 3b and c present the trajectory and speed for an example from one trial, for the movement marked by small white square in Fig. 3a. Note the very fast initial speed (Fig. 3c), just after the vBot released its hold on participant’s hand at the distal start location. This participant’s difficulty in progressing towards the distal target and in controlling the rebound movement in the opposite direction reflects the high tonus of the elbow flexors.

The *Guide* map in Fig. 3a (right) also shows two regions of high motor impairment. As in the *Assist* map the two impairment regions are horizontally aligned on the map, indicated the same range of target locations (the vertical axis), and are again centred approximately 180^o^ apart of each other in movement direction (horizontal axis). Yet, the directional tuning of these regimes is somewhat shifted relative to the directional tuning of the impairment regimes in the *Assist* map: the white and black squares lie between the regions of high *Guide* forces

Figure 5b  presents impairment maps of another chronic stroke participant (participant 2; right hemiparesis, 1 year after a stroke of the left hemisphere; UL F-M score 12/66), taken before and after 15 training sessions.

Prior to training (i.e. at a baseline session), both *Assist* and *Guide* maps show a double-blob pattern (Fig. 5b top row; note that the two separate red areas in the *Assist* map actually belong to a single region, given the angular map coordinates), similar to that of Participant 1 (Fig. 3a). However, the baseline maps of Participant 2 differ in the orientation and locations of the high impairment regions. For example, in the *Assist* map of Participant 2, the positive, red, impairment region includes target locations that are closer to the body (at the top half of the map, >180^o^), which are much less prominent for Participant 1. In addition, for Participant 2 the positive region includes movements which show impairment of internal rotation (starting directions between 0^o^-90^o^), and less impairment of external rotation movements (starting directions between 180^o^-270^o^); Participant 1 showed the opposite trends. Moreover, unlike the vertical nature of the regions in the *Assist* map of Participant 1, which reflects a relatively consistent range of impaired movement directions across a wide range of (distal) target locations, the positive impairment region of Participant 2 shows a diagonal pattern, indicating impairment in movement direction that co-varies with target location. Finally, for Participant 2, the positive red region (indicting slowness in moving) dominates in extent and magnitude. For Participant 1 the negative, blue, regions that indicate abnormally fast movements, dominate (with peak resisting forces ≥8N).

### Utilizing the map for individualized training

Apart from quantifying motor performance, we are using the performance maps to systematically and individually select movement conditions for training. Here we demonstrate their use in applying the SG principle of individualized selection for Participant 2.

Figure 5a  presents the baseline (pre-training) PM2 and PM3 performance maps (see the Methods section and the additional file [Additional file 1, ‘PM2 and PM3 maps’] for justification for using PM rather than force maps). Movement conditions that were selected for the first week of training are indicated on these maps by small black ‘x’ and white ‘+’ symbols, respectively. The PM2 and PM3 map patterns correspond closely to the baseline *Assist* and *Guide* force maps, respectively, (Fig. 5b, top row; note that positive red values in PM maps indicate better than expected motor performance and negative blue values indicate motor impairment; thus the polarity of the PM2 map is opposite to the *Assist* map). Indeed, the selected conditions are located at regions of steep transitions from higher to lower performance, regardless of the mapped parameters (PM2 or *Assist*, PM3 or *Guide*). Note also that while the regions differ between the PM2 and PM3 maps (in a similar fashion to the differences between the *Assist* vs. *Guide* maps) this difference between the two PM maps leads to different conditions being selected for training (compare the locations of the black ‘x’ vs. white ’+’ symbols in Fig. 5b). We return to this in the discussion.

Comparing the baseline versus post-training *Assist* and *Guide* maps in Fig. 5b (top vs. bottom maps, respectively) reveals the effect of the 15 training sessions on Participant 2’s reaching performance. Overall, the magnitude of assistance and guidance by the robot decreased, indicating improved performance. This is evident visually as shrinkage of the impairment regions in each of the maps. It is also evident quantitatively, with a decrease in mean impairment levels for each map, accompanied by a decrease in the standard deviation across the map, indicating flatter maps post-training (Table 1). Figure 5c summarises the pattern of change from baseline to post-training. As can be seen, the major improvement (blue areas, reduced forces) occurred in or near the regions selected for training. Still, there was some limited increase in *Guide* force, mostly around targets located at 90^o^ for movement directions between 270^o^-330^o^ (i.e. reaching diagonally left, toward distal targets across the body midline). This need for increased guidance was balanced by some reduction in the *Assist* force (indicated improved speed) in that region. This might suggest a speed-accuracy trade-off, pointing to a need for some future adjustments to the SG principle. However, this is beyond the scope of this report.

**Table 1:** Overall change of reaching movement impairment levels following training

	*Assist* map	*Guide* map
Change in mean force	-25.7%	-14.8%
Change in S.D. of force	-17.9%	-27.0%

The values represent the reduction in the mean force levels (i.e. improvement) across the Assist and Guide maps (before smoothing interpolation), recorded after 15 sessions of training, compared to baseline (Participant 2 only); the reduction in standard deviation of forces across the maps reflects a flattening of the performance maps

## Discussion

We have presented a novel method for the systematic mapping of motor performance and/or impairment of planar reaching movements across a workspace. We demonstrated the use of the method for identifying regions of movement impairment, in individuals with upper limb hemiparesis due to a stroke, and how it can be applied as a basis for performance-based selection of training movements for rehabilitation. The advantages of the performance mapping principle are that (1) mapping spans a wide region of the reaching movement conditions, and is fine-grained, potentially allowing high sensitivity to patterns of motor impairment, which may be overlooked by coarser clinical diagnostic scores (2) it is reasonably quick and easy to produce and to interpret, and (3) the maps’ coordinates (movement and target directions) relate to established basic elements of movement coding [22–27]. The mapping principle can be easily applied to any quantifiable scalar measures of reaching performance – behavioural metrics (e.g. kinetics and kinematics), physiological metrics (e.g. EMG; not demonstrated here), or impairment metrics (amount of assistance provided et cetera). Thus, that principle allows direct comparisons between maps of different modalities, for example EMG and kinetics. Here we demonstrated the mapping of arm movement impairment for individuals who had stroke. However, the method is likely to be beneficial also for profiling upper limb motor impairment as a result of other conditions including multiple sclerosis, cerebral palsy, or trauma.

The 2D mapping of motor performance is conducted across movement conditions defined by intended direction and target location, allowing mapping of a range of movements. The mapped movement conditions were confined to fixed start-to-target displacements and to targets that were located equidistant from a pre-defined centre location. For feasibility of testing and for ease of interpretation, the proposed method is limited to map reaching movements across a single 2D horizontal plane. These are typical of the training tasks used in end-effector robot-mediated UL therapy (e.g. [16]). Mapping motor performance of reaching movements across a full 3D range of movement, and across different target locations is possible in principle, but testing motor performance across the full 3D volume would be time consuming in a rehabilitation setting. Yet, if needed and if time allowed, multiple planar 2D maps could be prepared to approximate a full 3D map.

There have been few other attempts to develop systematic and fine profiling of arm motor impairment across a range of movement conditions, based on quantifiable motor performance measures. An early attempt by Kamper et al. [32] to map kinematic parameters of frontal reaches across a range of target locations and movement directions showed limited success in revealing individual patterns of impairment variation across the workspace. The success of our mapping method in revealing clear patterns of impairment suggests that the lower sensitivity of Kamper et al’s method might be related to the limited sensitivity of the performance measurements, perhaps due to the frontal planar arrangement of the targets and the lack of anti-gravity arm support, likely leading performance to be dominated by the challenge of opposing gravity [33]. High performance variability due to loose constraints on the movement trajectory may be another contributor to the limited sensitivity of their approach. Recently, a preliminary --but more sophisticated - approach for mapping performance distribution has been suggested [34], though is not yet utilizable as a tool for assessment of motor impairment.

Our mapping method is simple to use. This was demonstrated here in the maps of two participants (Figs. 3a and 5b), showing simple and clear impairment profiles, where impaired movements were concentrated in two map sub-regions. At the same time, the mapping exposed individual variation in the extent, location and orientation of the high impairment regions, emphasizing the potential importance of individualizing the selection of training conditions. Note, though, that the optimal exploitation of the performance map data for individualized therapy still needs to be demonstrated. We will present the results of a randomised and controlled study, comparing the steepest gradient-based selection of trained movements with more common “centre-out” training in one of our next publications.

Besides its potential utility as a basis for individualized UL therapy, the mapping method may contribute an added diagnostic utility. For example, mapping motor impairment across larger populations of people with stroke may allow the classification of their motor impairment, based on similarities in their maps’ patterns. Finding a few canonical motor impairment characteristics could then reduce the time spent on the full profiling of individuals’ impairment. In other words, a smaller number of probe locations might serve to discriminate and classify performance.

Another potential benefit of the mapping method is that it allows easy comparisons between maps of different impairment measures within and even between subjects. Such comparisons may potentially provide insightful information about causal relationships between different motor impairment factors. For example, high correlation between movement impairment maps and EMG maps (e.g. mapping levels of activity in the major arm muscles during the initiation of each reaching movement) may highlight the potential underlying roles of different muscles in different impairment patterns.

Some further improvements may be possible to optimize the principle of map-based selection of practiced movements. For instance, it is currently not clear whether combining selections based on both performance maps (as we have done, in our case selecting from PM2 and PM3 in proportion to the performance deficit each map showed) would be more beneficial than focusing on the one map that showed more impairment. Answering this requires further study, which we hope to take in the future. In addition, we have restricted the workspace to two dimensions, and the area, while covering much of the space involved in everyday hand action [35], is by necessity small compared to the full range of upper limb movement. However, as we will report in a subsequent paper, there is evidence of generalization of the training in these robotic environments to everyday actions, with improvement in clinical rating scores.

Theoretically, our mapping principle does not necessitate the use of a robot manipulandum and other movement measurement systems might be more affordably utilized to assess motor impairment, as long as they can provide quantifiable measures of motor performance. However, we do believe that robot-assisted forces are likely to provide sensitive measures of motor impairment – especially when impairment is more severe. Without the robotic assistance, motor impairment above some critical functional limit would block the participant’s ability to move, leading to a ‘floor effect’ in the measured performance, and so jeopardise the sensitive selection and quantification of training. This limitation should be considered when selecting the metrics of motor performance or impairment to be mapped.

## Conclusions

Our novel computerized mapping method is a feasible and simple approach for profiling upper limb performance across a wide movement workspace. It outlines regions of motor impairment in a clear way, allowing comparisons of impairment patterns within individuals, and between groups and can allow comparison of different motor impairment/performance measures. The performance maps can be utilized as a basis for individually-tailored therapy. Specifically, our mapping method allows for selection of training movement conditions that can be updated as rehabilitation progresses, dynamically tracking the changing performance of each participant. Comparison of individualized versus standardized training regimes is underway – but this is beyond the scope of the current report.

## Additional file

Additional file 1: Methods - supplemental details (text and a figure). This file contains further details about the robot-assisted reaching task, the robot assistance algorithm, robot-assistance parameter tuning, the definition of PM2 and PM3 parameters and their maps. (PDF 529 kb)

## Abbreviations

*Assist*: parameter measuring the overall amount of robotic force provided along the start-to-target axis during the full trial; ‘*Assist* force’ refers to the force provided at each moment along that axis.
F-M: Fugl Meyer clinical assessment of motor impairment
*Guide*: parameter measuring the overall amount of robotic force provided normal to the start-target axis.; ‘*Guide* force’ refers to the force provided at each moment along that axis.
PM2 & PM3: Performance measure parameters, adopted from [16], measuring the ability to move and aim, respectively (see Additional file for further details [Additional file 1, ‘PM2 and PM3 parameters’]).
SG: Steepest gradients
UL: Upper limb
vBot: robotic manipulandum used in the study

## References

[1] Dobkin BH (1997). Impairments, disabilities, and bases for neurological rehabilitation after stroke. J Stroke Cerebrovasc Dis.

[2] Lai SM, Studenski S, Duncan PW, Perera S (2002). Persisting consequences of stroke measured by the Stroke Impact Scale. Stroke.

[3] Lawrence ES, Coshall C, Dundas R, Stewart J, Rudd AG, Howard R, Wolfe CD (2001). Estimates of the prevalence of acute stroke impairments and disability in a multiethnic population. Stroke.

[4] Langhorne P, Coupar F, Pollock A (2009). Motor recovery after stroke: a systematic review. Lancet Neurol.

[5] Marchal-Crespo L, Reinkensmeyer DJ (2009). Review of control strategies for robotic movement training after neurologic injury. J Neuroeng Rehabil.

[6] Pollock A, Farmer SE, Brady MC, Langhorne P, Mead GE, Mehrholz J, van Wijck F: Interventions for improving upper limb function after stroke. In: Cochrane Database Syst Rev*.* edn.; 2014: CD010820. 10.1002/14651858.CD010820.pub2 PMC6469541 25387001

[7] Van Peppen RP, Kwakkel G, Wood-Dauphinee S, Hendriks HJ, Van der Wees PJ, Dekker J (2004). The impact of physical therapy on functional outcomes after stroke: what's the evidence?. Clin Rehabil.

[8] Kwakkel G, Wagenaar RC, Twisk JW, Lankhorst GJ, Koetsier JC (1999). Intensity of leg and arm training after primary middle-cerebral-artery stroke: a randomised trial. Lancet.

[9] Lo AC, Guarino PD, Richards LG, Haselkorn JK, Wittenberg GF, Federman DG, Ringer RJ, Wagner TH, Krebs HI, Volpe BT, et al. Robot-assisted therapy for long-term upper-limb impairment after stroke. 2010;362 10.1056/NEJMoa0911341 PMC5592692 20400552

[10] Norouzi-Gheidari N, Archambault PS, Fung J (2012). Effects of robot-assisted therapy on stroke rehabilitation in upper limbs: systematic review and meta-analysis of the literature. J Rehabil Res Dev.

[11] Winstein C, Wing AW, Whitall J. Motor control and learning principles for rehabilitation of upper limb movements after brain injury. In: Grafman JR, Robertson IH, editors. Handbook of Neuropsychology, vol. 9. 2nd ed. New York: Elsevier Science; 2003. p. 77–137.

[12] Lohse KR, Lang CE, Boyd LA (2014). Is more better? Using metadata to explore dose-response relationships in stroke rehabilitation. Stroke.

[13] Lang CE, Lohse KR, Birkenmeier RL (2015). Dose and timing in neurorehabilitation: prescribing motor therapy after stroke. Curr Opin Neurol.

[14] Han C, Wang Q, Meng PP, Qi MZ (2013). Effects of intensity of arm training on hemiplegic upper extremity motor recovery in stroke patients: a randomized controlled trial. Clin Rehabil.

[15] Kwakkel G, Kollen BJ, Krebs HI (2008). Effects of robot-assisted therapy on upper limb recovery after stroke: a systematic review. Neurorehabil Neural Repair.

[16] Krebs HI, Palazzolo JJ, Dipietro L, Volpe BT, Hogan N (2003). Rehabilitation robotics: Performance-based progressive robot-assisted therapy. Auton Robot.

[17] Burgar CG, Lum PS, Scremin AM, Garber SL, Van der Loos HF, Kenney D, Shor P (2011). Robot-assisted upper-limb therapy in acute rehabilitation setting following stroke: Department of Veterans Affairs multisite clinical trial. J Rehabil Res Dev.

[18] Liao WW, Wu CY, Hsieh YW, Lin KC, Chang WY (2012). Effects of robot-assisted upper limb rehabilitation on daily function and real-world arm activity in patients with chronic stroke: a randomized controlled trial. Clin Rehabil.

[19] Guadagnoli MA, Lee TD (2004). Challenge point: a framework for conceptualizing the effects of various practice conditions in motor learning. J Mot Behav.

[20] Alaverdashvili M, Foroud A, Lim DH, Whishaw IQ (2008). Learned baduse limits recovery of skilled reaching for food after forelimb motor cortex stroke in rats: a new analysis of the effect of gestures on success. Behav Brain Res.

[21] Sanger TD (2004). Failure of motor learning for large initial errors. Neural Comput.

[22] Sergio LE, Hamel-Paquet C, Kalaska JF (2005). Motor cortex neural correlates of output kinematics and kinetics during isometric-force and arm-reaching tasks. J Neurophysiol.

[23] Kettner RE, Schwartz AB, Georgopoulos AP (1988). Primate motor cortex and free arm movements to visual targets in three-dimensional space. III. Positional gradients and population coding of movement direction from various movement origins. J Neurosci.

[24] Lacquaniti F, Guigon E, Bianchi L, Ferraina S, Caminiti R (1995). Representing spatial information for limb movement: role of area 5 in the monkey. Cereb Cortex.

[25] Hamel-Paquet C, Sergio LE, Kalaska JF (2006). Parietal area 5 activity does not reflect the differential time-course of motor output kinetics during arm-reaching and isometric-force tasks. J Neurophysiol.

[26] Ghez C, Scheidt R, Heijink H (2007). Different learned coordinate frames for planning trajectories and final positions in reaching. J Neurophysiol.

[27] Hudson TE, Landy MS (2012). Motor learning reveals the existence of multiple codes for movement planning. J Neurophysiol.

[28] Engineer ND, Engineer CT, Reed AC, Pandya PK, Jakkamsetti V, Moucha R, Kilgard MP (2012). Inverted-U function relating cortical plasticity and task difficulty. Neuroscience.

[29] Howard IS, Ingram JN, Wolpert DM (2009). A modular planar robotic manipulandum with end-point torque control. J Neurosci Methods.

[30] Ferraro M, Palazzolo JJ, Krol J, Krebs HI, Hogan N, Volpe BT (2003). Robot-aided sensorimotor arm training improves outcome in patients with chronic stroke. Neurology.

[31] Fasoli SE, Krebs HI, Stein J, Frontera WR, Hughes R, Hogan N (2004). Robotic therapy for chronic motor impairments after stroke: Follow-up results. Arch Phys Med Rehabil.

[32] Kamper DG, McKenna-Cole AN, Kahn LE, Reinkensmeyer DJ (2002). Alterations in reaching after stroke and their relation to movement direction and impairment severity. Arch Phys Med Rehabil.

[33] Beer RF, Dewald JP, Dawson ML, Rymer WZ (2004). Target-dependent differences between free and constrained arm movements in chronic hemiparesis. Exp Brain Res.

[34] Huang FC, Patton JL (2016). Movement distributions of stroke survivors exhibit distinct patterns that evolve with training. J Neuroeng Rehabil.

[35] Ingram JN, Kording KP, Howard IS, Wolpert DM (2008). The statistics of natural hand movements. Exp Brain Res.

[36] Flash T, Hogan N (1985). The coordination of arm movements: an experimentally confirmed mathematical model. J Neurosci.

