# Supplementary material for: Mapping upper-limb motor performance after stroke - a novel method with utility for individualized motor training
Source: J Neuroeng Rehabil. 2017 Dec 6;14:127. doi: 10.1186/s12984-017-0335-x (PMC5718133; doi:10.1186/s12984-017-0335-x)
Supplement: Additional file 1: — Methods - supplemental details (text and a figure). This file contains further details about the robot-assisted reaching task, the robot assistance algorithm, robot-assistance parameter tuning, the definition of PM2 and PM3 parameters and their maps. (PDF 529 kb) [file 12984_2017_335_MOESM1_ESM.pdf]

METHODS - SUPPLEMENTAL DETAILS

(Manuscript: "*Mapping upper-limb motor performance after stroke– a novel method with utility for individualized motor training"*

By O Rosenthal, AM Wing, J Wyatt, D Punt and RC Miall)

Task and settings

The robot-assisted task procedure, stimulus presentations and data acquisition were programmed in C++ using a vBot code library[1]. Parameter tuning, performance mapping, and selection of training movements were performed in Matlab (R2007b, Mathworks Inc. Communication between the two programmes was achieved by shared access to data files. During training sessions performance data were exported from the vBot after every block of 20 trials, and read, processed and plotted within Matlab to provide summary feedback. A subset of the performance-based selection of movement conditions (start and target locations) were then exported from Matlab for reading into the vBot control program, for the next block of 20 trials. In the performance mapping sessions, performance data was exported at the end of the session to be processed and summarized Matlab program, with no ongoing summary feedback.

During the robot-assisted sessions participants were seated on a wheelchair (wheels locked), gripping the handle of a planar robotic manipulandum (vBot[1]) with their affected hand (Figure 1A in the main article). Upright sitting posture was ensured through a cushion behind the participant's back and by a butterfly harness worn around the participant's trunk and fastened to back of the seat. The hand grip was ensured by a special glove (Active Hands; http://www.activehands.com), which also helped to stabilize the wrist. The arm was supported against gravity using a mechanical dynamic arm support to the forearm (SaeboMAS®; http://www.saebo.com/), with the level of gravity assistance individually adjusted to the individuals' need at the baseline session and then maintained at that level.

The participants leaned their forehead on a headrest and viewed a horizontal mirror, approximately 10 cm below eye level (Figure S1). A 32 inch computer screen was mounted horizontally 24 cm above the horizontal mirror so that the participant could see the virtual image of the display congruent with the plane of motion of the vBot handle. The mirror concealed the handle and the participant's hand from direct view, but the handle location was accurately indicated on the display by a cursor (a small red disc 0.3 cm radius). The participant's body midline was aligned with the centre of the display. The height of the entire workstation could be adjusted individually.

The two degree-of-freedom robotic manipulandum permits planar movements of its end-point handle, with minimal back-drive friction and inertia, across a workspace of approximately 80x45cm[1]. A foot-operated safety switch was continuously depressed by the experimenter to activate the robot when the participant was ready for each session; release of the footswitch immediately cancelled any forces generated by the motors. The vBot handle position, velocity and all applied forces (motor torques, limited to maximum of 100N) were recorded at high precision (sampling rate 1000 Hz).

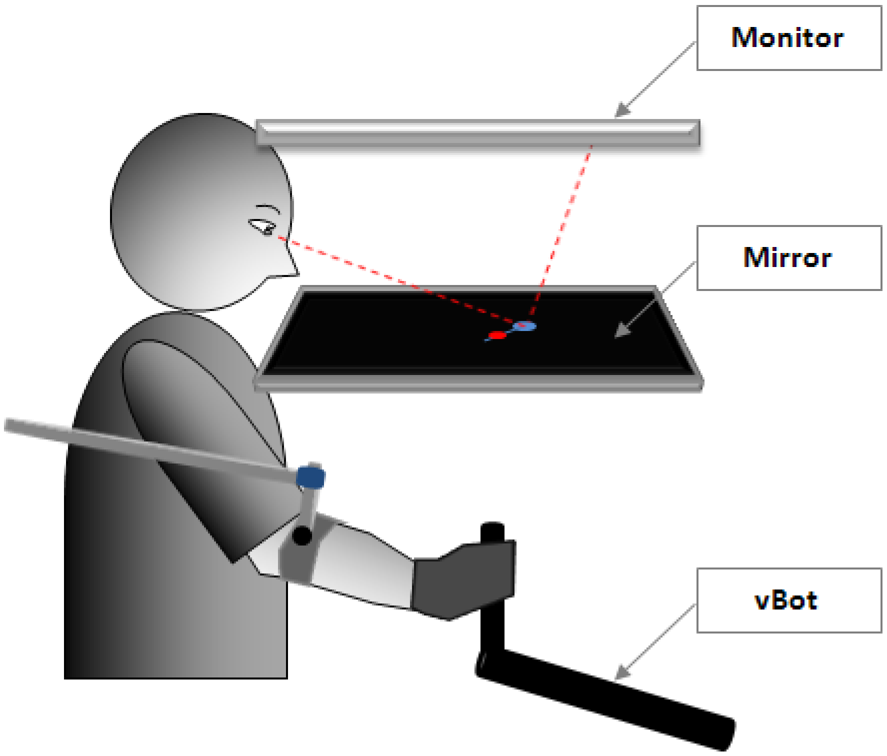

Figure S1. Schematic illustration of the relative positions of the computer monitor, mirror, vBot and the participant. Visual stimuli were generated on the monitor and reflected by the mirror to the participant's eyes (shown are the on-screen indicators of the hand (vBot's handle; red) and target (blue) position).

At the beginning of each trial a start position was indicated by a small white disk (0.3 cm) and the vBot gently guided the participant's hand towards it using a minimum-jerk-based impedance controller[2]. Once the participant reached the start position the robot maintained the hand there with a spring-like resistance until a target was presented 5 cm distant indicated by a blue disk (0.5 cm radius) and a thin blue line connected the start and target position. The vBot then released hold of the hand over 100 ms. The participant attempted to move the handle to a target within an allotted time (set individually, see below). During the trial the vBot provided forces as needed to assist and guide the hand to the target. At the end of the trial the target disappeared and an animated explosion provided feedback about the reach accuracy, where the final explosion radius indicated the proximity of the end hand's location to the target.

Robot assistance algorithm:

We adopted a revised version of the assistance algorithm that was developed by Krebs et al.[3]. At any moment during the attempted movement the robot could provide forces along two orthogonal axes:

1. Assist forces were provided along the start-to-target axis. The magnitude and sign of *Assist* force at any moment depended on (i) the difference between the actual and expected progression along the time-constrained minimal-jerk-based trajectory[2] and (ii) whether or not the attempt to move was compromised by high muscle tone.

The allotted movement duration was set individually during an initial tuning session (see below). At any moment the actual hand location was compared to the expected location (based on the minimum jerk trajectory defined by that duration). At any moment, if

progression towards the target was delayed (compared to expected), a damped spring-like *Assist* force was applied towards the target. The stiffness constant depended on whether or not the initial delay exceeded a criterion indicating high muscle tone (12 and 6 Ncm^{-1}, respectively). High muscle tone could lead to very strong resistance to the intended movement (Figures 3B and 3C, left, main article). On the other hand high tone could also lead to very fast, rebound-like movement typically from an extended elbow posture when the robot, holding the hand at the start position, released the hold to initiate the trial (Figures 3B and 3C, right, and Figure 4, main article). If the initial movement towards the target was abnormally fast then a negative *Assist* force acted to slow the movement and promote its expected smooth progression (stiffness constant: 12 Ncm^{-1}). No *Assist* force was provided for movements which were faster than allotted time but still within the range of normal voluntary movements (i.e. which would lead to 5cm displacement within 200ms or longer; estimated using minimum jerk). Additionally, target overshoots were also restrained by the robot.

2. Guide forces were provided lateral to the start-to-target axis and their magnitude at any moment depended on the extent of the deviation from the intended straight-line path, imposing damped spring-like behaviour. The *Guide* force stiffness, k_{sw}, was set individually in the first parameter tuning session (see below), except in case of abnormally fast movements where it was set to 12 Ncm^{-1}.

The robot controllers were triggered upon the initiation of the movement, or 2 seconds after target onset in rare occasions when the participant failed to start. In all cases, to minimize the risk of transient harmful movements a weak damping was used during the intra-trial-interval, and the introduction and termination of assistive and guiding forces were ramped up over a period of 100 ms.

Robot-assistance parameter tuning session

The allotted movement time (t_a) and the *Guide* force's stiffness constant (k_{sw}) were set individually during an initial tuning session, adopting Krebs et al's staircase method[3]. Parameters were adjusted after each cycle of 32 trials. Movements compromised by high muscle tone were excluded from the adjustment algorithm. Briefly, the rule for adjustment of t_a in cycle *n+1* was: $t_a(n+1) = t_a(n) + 2.0PM2(n)$ and the rule for adjustment of k_{sw} was: $k_{sw}(n+1) = k_{sw}(n) + 3.0PM3(n)$. PM2 and PM3 are performance parameters (defined below).

For the tuning session the full set of 64 movement conditions was divided to two subsets of 32 movements to allow staircase-wise parameter adjustment. Each sub-set included all 8

target locations but only 4 relative start locations (0°, 90°, 180° and 270° for one subset, and 45°, 135°, 225° and 315° for the other subset); 32 movements were sampled pseudo-randomly from one subset, then from the other subset and so forth.

Usually, tuning of the assisted parameters approached a plateau within a single session (10 cycles, or 320 trials). In rare cases there was a need of an extra session. Following the tuning session, the robot-assisted parameters (t_a and k_{sw}) were fixed for the particular individual. All subsequent robot-assisted sessions (performance mapping and/or training) then used these fixed parameters.

PM2 and PM3 parameters

The definition of PM2 and PM3 parameters was adopted from [3]. Here we provide only a brief description of these parameters, since the focus of this paper is on performance mapping procedure rather than on a specific performance measure. The PM2 and PM3 parameters measure the ability to move and to aim, relative to individually-set performance criterion, respectively. Negative PM values indicate worse than performance criterion and positive values indicates exceeding (better than) the performance criterion, set individually in the initial tuning session. Specifically, when movement towards the target is slower than expected for the particular participant (or abnormally fast) PM2 is negative and reflects the average robot power ($\sum_{i=1}^{N}[F_y(i)\dot{y}(i)]$) in the direction of start-target axis (*y*). The index *i* indicates the time point during the individually-set allotted movement time t_a. Hence $F_y(i)$, is the *Assist* force provided along the start-target axis at time point *i*, and $\dot{y}$ is velocity. On the other hand, when the movement is controllable and faster than expected (thus assistance is not required), PM2 is positive and defined such that it reflects the total difference between the momentary expected and actual movement displacement along the trajectory ($\sum_{i=1}^{N}[y(i) - y_{m.j}(i)]$). Here $y_{mj}(i)$ is the expected progress towards the target at time point *i*, given the allotted time t_a assuming a minimum jerk trajectory (Flash and Hogan 1985). PM3 depends on the difference between root-mean-square deviation ($\sqrt{\frac{1}{N}\sum_{i=1}^{N}x(i)^2}$) along the axis normal to the start-to-target (x) and the tolerated deviation, scaled by the individually set stiffness constant (k_{sw}) of the *Guide* force. Similarly to PM2, negative PM3 values indicate impaired aiming and positive values indicate better than expected values.

Performance mapping sessions were used for creating PM maps that then allowed selection of movements in the following training sessions (see below).

Note that since both PM2 and PM3 parameters are defined relative to individually-set parameters, they are not directly comparable across subjects. Note also that PM3 is a purely kinematic measure and therefore may be biased due to the fact that the movement is assisted; in other words the kinematics are affected by the robot guidance and assistance

Assist* and *Guide* vs *PM2 and PM3 maps:

The *Assist* and *Guide* parameters are useful portrayals of impairment, with minimal performance-assistance confounding issues (see main article). Note, though, that these parameters are not sensitive to unimpaired performance (both measures are limited to zero level when no assistance is needed). This makes these measures unsuitable for analysis that requires computing a gradient. Hence, an alternative measure of performance was employed for the selection of training conditions, and for that purpose we used PM2 and PM3 as alternatives to the *Assist* and *Guide* measures, respectively. However, there are several consequences of using these parameters to characterise performance. First, they measure performance relative to individually adjusted parameters (t_a for PM2 and k_{sw} for PM3) and are thus not fully comparable across individuals. Second, PM3 is purely a kinematic measure – and hence it is confounded by the robot assistance. Finally, PM parameters are less intuitive than the force measures (*Assist* and *Guide*). For these reasons, when possible, we recommend using *Assist* and *Guide* to describe performance, and PM2 and PM3 to select trained movements.

Impairment-based proportion of movement selection based on PM2 and PM3 maps

Individuals differ in their ability to aim and to complete movements. Hence, the selection of training movements should reflect the performance map that best captures their individual impairment. Accordingly, the number of training conditions selected from each map (PM2 or PM3) was weighted in proportion to the mean performance over the worst 25% of each map, such that more training conditions were selected from the map that showed worse motor performance. Specifically, for each map PM_i (i=2,3), the mean performance $\overline{PM_i^{25\%}}$ was computed across the lowest 25% of the scores of the map. The $\overline{PM_i^{25\%}}$score was then ranked as mild (W_{PMi}=0), moderate (W_{PMi}=1) or severe (W_{PMi}=2) (see[1]). Movements to form a training set were then chosen at random from two sets of 102 movements selected based on the steepest gradients of PM2 and PM3, to satisfy the numerical weighting:

$$N_{PM2} = \frac{W_{PM2}}{W_{PM2}+W_{PM3}} 102 \;\; and \;\; N_{PM3} = \frac{W_{PM3}}{W_{PM2}+W_{PM3}} 102 \, ,$$

In the possible case where both maps show mild impairment (hence W_{PMi}=0) the movements in the training set would be sampled equally and randomly from each of the N_{PM2} and N_{PM3} movements. None of our participants showed this outcome.

REFERENCES

1. Howard IS, Ingram JN, Wolpert DM: **A modular planar robotic manipulandum with end-point torque control**. *J Neurosci Methods* 2009, **181**(2):199-211.
2. Flash T, Hogan N: **The coordination of arm movements: an experimentally confirmed mathematical model**. *J Neurosci* 1985, **5**(7):1688-1703.
3. Krebs HI, Palazzolo JJ, Dipietro L, Volpe BT, Hogan N: **Rehabilitation robotics: Performance-based progressive robot-assisted therapy**. *Auton Robot* 2003, **15**(1):7-20.

---

[1] $W_{PMi} = 0$: $\overline{PM_i^{25\%}} \geq -0.01$;
$W_{PM2} = 1$: $-0.01 > \overline{PM2^{25\%}} \geq -0.4$
$W_{PM3} = 1$: $-0.01 > \overline{PM3^{25\%}} \geq -0.5$
$W_{PM2} = 2$: $-0.5 > \overline{PM2^{25\%}}$
$W_{PM3} = 2$: $-0.4 > \overline{PM3^{25\%}}$
